# 3D-Printed core-shell tablet for effective oral delivery of AT-MSC secretome in inflammatory bowel disease therapy

**DOI:** 10.1007/s13346-025-01932-7

**Published:** 2025-08-08

**Authors:** Elena Munoz-Perez, Edorta Santos-Vizcaino, Alvaro Goyanes, Abdul W. Basit, Rosa Maria Hernandez

**Affiliations:** 1 NanoBioCel Research Group, Laboratory of Pharmaceutics, School of Pharmacy, University of the Basque Country (UPV/EHU), Paseo de la Universidad 7, Vitoria-Gasteiz, 01006 Spain; 2 Biomedical Research Networking Centre in Bioengineering, Biomaterials and Nanomedicine (CIBER- BBN), Institute of Health Carlos III, Madrid, 28029 Spain; 3 Bioaraba, NanoBioCel Research Group, Vitoria-Gasteiz, 01006 Spain; 4 Departamento de Farmacología, Farmacia y Tecnología Farmacéutica, Facultad de Farmacia, Instituto de Materiales (iMATUS) and Health Research Institute of Santiago de Compostela (IDIS), Universidade de Santiago de Compostela, Santiago de Compostela, 15782 Spain; 5 Department of Pharmaceutics, UCL School of Pharmacy, University College London, 29-39 Brunswick Square, London, WC1N 1AX UK

## Abstract

The complexity of inflammatory bowel disease (IBD) and its pharmacological management has driven research to explore novel treatment options for this condition. Among the emerging therapies, Mesenchymal Stromal Cells (MSCs) have proven to be effective, showing strong immunomodulatory properties. However, the efficiency of direct MSC administration has been questioned due to their short half-life and risk of rejection. As an alternative, MSCs secretome, containing the beneficial paracrine effectors of MSCs, is being explored as a promising cell-free therapeutic tool. However, the oral delivery of this secretome is complex and presents a significant technological challenge. Additionally, the personalization of secretome-based therapies is essential, as it is a costly treatment that requires dose customization for each patient. This study introduces a novel approach for the oral delivery of adipose-tissue derived MSC secretome (AT-S) using 3D-printed core-shell tablets. Remarkably, lyophilized secretome (LpAT-S) was used during the study to improve the manageability and stability of the secretome. The 3D printed system proved to be capable of protecting secretome proteins from gastric degradation, offering an unprecedented possibility for the oral administration of secretome therapies. Overall, this study shows that 3D printing offers a promising, patient-friendly solution for the oral administration of MSC secretome for the first time, aligning with precision medicine goals to provide tailored therapies for IBD.

**Graphical abstract:**

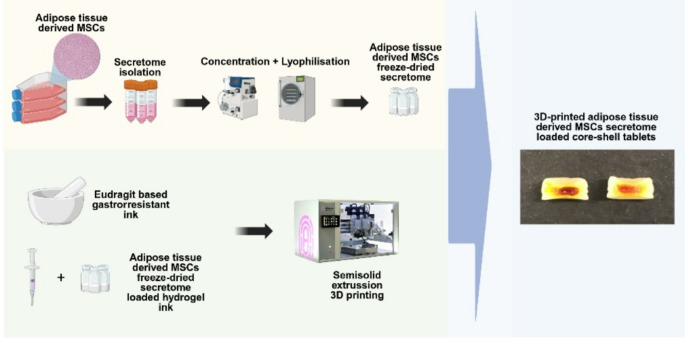

**Supplementary Information:**

The online version contains supplementary material available at 10.1007/s13346-025-01932-7.

## Introduction

The pharmacological management of Inflammatory Bowel Disease (IBD) is intricate. The main treatments on IBD focus on relieving symptoms and usually involve corticosteroids, biological therapies, or strong immune system suppressors for severe cases. However, resistance to these drugs and their side effects greatly reduce their efficacy [1, 2]. Consequently, research efforts underway to develop novel therapeutic strategies for IBD. Among others, the use of mesenchymal stromal cells (MSCs) for the management of IBD is considered a well-known stated therapeutic approach [3]. The immunomodulatory capacity of MSCs and their ability to modulate the immune response, brand MSCs as a novel therapeutic weapon in IBD [4, 5]. However, the limitations and risks attributed to the administration of MSCs in vivo (i.e. tumorigenicity, thromboembolism, rejection risks etc.) have caused a paradigm shift that seeks the implementation of “cell-free therapies” [6–8]. Thus, the paracrine mediators secreted by MSCs, named secretome, have been the novel research target. Notably, the MSCs secretome (MSCs-S) has been linked to induce most of the beneficial effects observed in MSCs therapies, regulating the exaggerated or irregular immune responses seen in IBD by controlling cellular behavior in both innate and adaptive immunity cells [5, 9].

Therefore, in recent years, there has been a vigorous pursuit to control the delivery of secretome within drug delivery devices. Since then, research on secretome administration on IBD in vivo models has been focused on its intraperitoneal or intravenous direct injection or its rectal administration inside of soft vehicles such as hydrogels [10–14]. However, despite being one of the most demanded routes by patients, the oral administration of MSCs-S remains unaddressed. This is likely due to the significant challenges associated with administering MSCs-S orally. As a key point, gastric degradation of the components of MSCs-S is a worrying fact. Thus, ensuring the safe delivery of proteins and growth factors included on it, to the affected areas of the intestine and colon without degradation is of special relevance and highly complicated [5, 14].

Not only does the fragility of the secretome poses challenges, but its complex handling also presents technological limitations for its oral formulation [10]. Firstly, MSCs-S is typically obtained and delivered in liquid form, making it demanding to convert into conventional oral pharmaceutical forms like coated capsules or tablets that can withstand gastric conditions. Noteworthy, accurate dosing of the MSCs-S becomes limited, as large volumes of liquid cannot be easily administered protected, which further constrains the efficient treatment, requiring frequent and multiple dosing. Similarly, drug titration is particularly important when administering MSCs-S, given the lack of precisely established effective doses and the variability in both secretome composition and patient responses. Therefore, rapid titration in patients and dose adjustments within formulations are imperative to optimize treatment outcomes [5, 10, 12, 14, 15].

Considering this framework, it is marked that there is a striking need for the development of novel pharmaceutical dosage forms for the oral administration of secretome. These new formulations must not only shield the secretome from gastric degradation, but also enable rapid formulation in easily modifiable doses, allowing greater flexibility in administration and the production of cost-effective secretome based therapies for IBD [14].

In this context, the development of pharmaceutical forms must not only match but drive the innovation seen in modern therapeutics. To achieve this, cutting-edge manufacturing strategies that provide control and precision in drug delivery systems are essential. A key example of this innovation is the emergence of 3D printing technologies in pharmaceutical manufacturing. These cutting-edge techniques provide unparalleled versatility, allowing for the design of highly specialized dosage forms tailored to specific therapeutic needs, redefining the possibilities of drug delivery [16, 17].

Among the advantages of this technique, 3D printing allows exhaustive control over the production of therapies. It is usually performed in small batches, which allow better analysis and quality control of the formulations [18]. Likewise, small batch production reduces the waste of the excipients and drugs employed, which is of great importance in the case of MSCs-S based therapies in which the pharmaceutical economy is a key player. Furthermore, controlling the shape and size of dosage forms during printing allows greater control over the dosing of therapies and their drug release profile [19]. Thus, by modifying the size and shape of a formulation, its pharmacokinetics can also be tailored. As a main benefit, these modifications can be quickly applied, adjusting the digital model (CAD model) of the printed formulation to the needs of each patient and therapy requirements. Notably, 3D printing of medicines offers the possibility of making complex pharmaceutical dosage forms [20]. The personalized spatial combination of different pharma-inks allows the design of formulations that are difficult to manufacture with usual manufacturing techniques [21]. This allows the creation of compartmentalized pharmaceutical forms that can protect fragile drugs or combine multiple therapies into a single formulation, making treatments simpler for patients [22]. These 3D printing approaches are aligned with the objective of protecting and effectively delivering MSCs-S and take a step further trough precision medicine.

Therefore, in this study, we combined the highly promising therapy of adipose-tissue derived MSCs-S (AT-S) with 3D printing techniques to create formulations suitable for oral delivery. As a key point, to make the AT-MSCs-S manageable and to simplify its formulation, a lyophilized AT-MSCs-S (LpAT-S) powder was employed for the whole development.

We employed the Semisolid Extrusion 3D printing (SSE) technique, which is particularly advantageous as it can be performed without high heating temperatures, thereby safeguarding the integrity of the secretome cytokines and growth factors against denaturation [23, 24].

This way, during the printing process of the core-shell tablets, two types of Pharma-inks were used and positioned in different areas of the formulation: one Pharma-ink based on Eudragit, a polymer commonly used for the coating of gastro-resistant formulations, which was printed as an external shell of the formulation; and another Pharma-ink containing the Lp-AT-S formulated inside of an alginate hydrogel, allowing its controlled dosing through extrusion inside of a protected central compartment. As a result, a core-shell shape AT-MSCs-S loaded tablet was obtained. Throughout the study, we successfully developed innovative Pharma-ink formulations and refined the printing method for advanced 3D-printed core-shell tablets (3DP core-shell tablets). The printed formulations were characterized in terms of shape, size, and internal structure, ensuring the reproducibility and precision of the cutting-edge 3D printing technique. Moreover, the release kinetics of the formulations were established through dissolution studies of fluorescein-loaded systems. We also demonstrated the remarkable ability of these 3DP core-shell tablets to safeguard secretome proteins from the gastric pH environment.

## Materials and methods

### LpAT-S production

#### AT-MSC culture and licensing

The cells used in this study were AT-MSCs (ATCC, PCS-500-011). Cells were cultured in Mesenchymal Stem Cell Basal Medium (ATCC^®^, Cat. No: PCS-500- 030™) supplemented with the Mesenchymal Stem Cell Growth Kit (ATCC^®^, Cat. No: PCS-500–040). Cells were cultured at 37 °C in a 5% CO2 / 95% air atmosphere until reaching 80–90% confluence. Afterwards, cells were overnight (16 h) licensed employing 60 ng/mL IFNγ and 60 ng/mL TNFα licensing culture media (ATCC^®^, Cat. No: PCS-500- 030™). After licensing, cells were maintained in serum-free non-supplemented media in order to allow secretome production for 48 h. Finally, secretome was collected and centrifuged at 2000 *g* for 10 min in order to remove cell debris. Finally, the secretome was freeze-dried and stored at -80 °C until usage.

### Pharma-ink preparation

Pharma-ink for the outer shell part of the formulations was prepared by mixing components with the help of mortar and pestle. Briefly, Eudragit L100-55 (Evonik^®^, Darmstadt, Germany) was thoroughly mixed with triethyl citrate (TEC) (Thermo Fisher Scientific, Waltham, MA, USA) and a hydroxyl propyl methylcellulose (HPMC) (Thermo Fisher Scientific, Waltham, MA, USA) 10% dispersion in order to create a white homogeneous paste (Safe shell ink). Accurate composition displayed on Table 1.

Pharma-ink for the inner core part of the formulation was formulated as an alginate hydrogel. Briefly, ultrapure sodium alginate (Novamatrix) was dissolved in distilled water at a concentration of 6% (w/v). For the formulation of fluorescein loaded 3DP core-shell tablets, the alginate solution was mixed using the double syringe method with a fluorescein solution containing 6% fluorescein (w/v) and calcium sulfate (Gibco, Thermo Fisher Scientific, Waltham, MA, USA) at a concentration of 60 mM. Consequently, a 2% (w/v) crosslinked alginate solution was obtained, containing 3% (w/v) fluorescein (Thermo Fisher Scientific, Waltham, MA, USA) (Table 2).

**Table 1:** Composition of the Eudragit based paste used to obtain the 3DP-core-shell tablets. Components expressed in % (w/w)

Pharma-ink	Eudragit L100-55 (%)	TEC (%)	HPMC (%)	Water (%)
Safe shell	41	20	4	35

**Table 2:** Composition of the alginate hydrogel ink employed for the internal core of the 3DP core-shell tablets. Secretome dose hydrogel employed for secretome-loaded formulations. Fluorescein contrast ink used for fluorescein loading of formulations. Components expressed in % (w/w)

Pharma-ink	Alginate (%)	Fluorescein (%)	Secretome (%)	Calcium sulphate (uM)	Water (%)
Secretome dose hydrogel	2	-	26.66	0,07	71.27
Fluorescein contrast	2	3	-	0,07	94.93

On the other hand, for secretome loaded 3DP core-shell tablets, the alginate solution was mixed using the double syringe method with a calcium sulfate solution at a concentration of 60 mM in which 40 mg of LpAT-S was dispersed. As a result, a 2% (w/v) crosslinked alginate solution was obtained containing 40 mg of LpAT-S inside (Table 2).

### Design of 3D-printed tablets

The software “123Design” was employed to design the 3D models of the tablets. For that purpose, a cylinder model of 10 mm width and 5 mm height was design. A hollow of 3 mm width and 6 mm height was included in the design. Repetier Host^®^ software was used for slicing. Manual programming was employed to settle the SSE cartridge switching in order to print the shell of the formulation with safe shell ink in and the core of the formulation with the hydrogel inks.

### Semi-solid extrusion 3D printing process

Pharma-inks were transferred into 20 mL syringes and placed into the pharmaceutical 3D printer M3DIMAKER 2™ (FABRX Ltd., London, UK). The shell of the 3D design was printed first employing safe shell ink. The core was printed employing fluorescein contrast ink or secretome dose hydrogel ink. The printer settings were stated as 3 mm/s extruding speed and 10 mm/s travelling speed. Shell number was stablished as 2 and infill percentage selected was 20% with a layer height of 1.2 mm. 16 G Conical nozzles were employed for printing. No temperature was employed during extrusion.

### Printlet characterisation

Because the dissolution profile characterization of the core-shell tablets was performed using a model molecule like fluorescein for tracking, the morphological characterization was conducted on both formulations: 3DP core-shell tablets loaded with secretome and 3DP core-shell tablets loaded with fluorescein.

#### Morphological evaluation

The diameter and height of the formulations were measured with a digital caliper (DigiMax^®^, Dublin, Ireland), and their weight was determined using a FisherBrand analytical balance (Fisher Scientific, Leicestershire, UK) (*n* = 10). The mean values were calculated, and the results were expressed as the mean ± standard deviation.

#### Scanning electronic microscopy (SEM) imaging

SEM images of the tablets were performed in order to analyze the structure of each of the outer and inner part of the core-shell tablets. A JSM-840 A Scanning Microscope (JEOL GmbH, Freising, Germany) was employed for the analysis. Differences in the internal structure of the crosslinked alginate hydrogels when loaded with secretome or blank was analyzed. 25 nm layer height gold coating of the samples was employed prior to visualization by means of a sputter coater. SEM analysis was performed at 5 kV using a backscattered electron detector/secondary electron detector.

#### Mass variation

Twenty 3D-printed core-shell tablets were weighted and the average mass was calculated. The mean values were calculated, and the results were expressed as the mean ± standard deviation.

#### Friability testing

Friability tester was employed for the assay (SBS Instruments, TDA − 1). As average mass of the core-shell tablets was below 650 mg a sample of whole tablets corresponding to 6.5 mg was carefully weight prior the assay. Then, tablets were introduced inside the drum and rotated 100 times using a speed of 25 rpm. Loose dust was brushed from the tablets and their final weigh was noted.

#### Hardness

A tablet hardness-testing instrument was employed for the determination (Pharma Test, PTB 311). A total of ten 3D-printed core-shell tablets were tested.

#### Humidity test

A moisture analyzer (Mettler Toledo, HB43) was employed for the test. Briefly, printlets were grind and 1 g powder of each printlet formulation was weighted. Weight loss in the printlet powder was noted after a heating period 15 min at 100 °C.

#### Disintegration time

To determine the disintegration time of the core-shell tablets European pharmacopeia requirements for gastro-resistant tablets were followed. Briefly, six units of each formulation were first immersed in 0.1 M hydrochloric acid for 2 h. The integrity of the formulations was checked after that period. Afterwards the disintegration test was performed in phosphate buffer pH 6.8 medium for 1 h and the disintegration time of the formulations was noted. Basket-rack disintegration apparatus (USP I SOTAX AT 7) at 37 °C was employed for the assay.

#### Dissolution testing

In order to test dissolution profile of the core-shell tablets, the release of fluorescein from the tablets was tested. Thus, 3DP core-shell tablets containing fluorescein contrast ink were printed and harden. Dissolution test was perfomed as described in the European pharmacopeia methods. USP 2 dissolution apparatus was employed for the assay (ERWEKA, Heusenstamm, Germany). Briefly, fluorescein loaded 3DP core-shell tablets (*n* = 6) were immersed in 750 mL, 0.1 M HCl in each dissolution vessel for two hours. Paddle rotation was stated in 75 rpm. After the acid state 250 mL of 0.2 M trisodium phosphate solution was added into each vessel and the pH was adjusted to 6.8 using NaOH or HCl solutions. Samples of 1.5 ml were taken and replaced with fresh media at time points: 0.5, 1, 1.5, 2, 2.5, 3, 4, 6 and 8 h. Fluorescein concentration in samples was determined employing UV-Vis spectrometry operating at 490 nm.

Fluorescein release data (Mt/M∞) was analyzed according to Zero order (1), Korsmeyer-peppas (2) and Peppas and Sahlin (3) equations:

1 $$\:Mt/M\infty\:=kt$$

2 $$\:Mt/M\infty\:=k{t}^{n}$$

3 $$\:Mt/M\infty\:=kd{t}^{m}+krt{2}^{m}$$

Where 𝑀𝑡/𝑀∞ is the Fluorescein released at time t (M ∞ corresponds to the 100% of fluorescein released), *k* is the zero-order release constant on Eq. 1 and the Korsmeyer kinetic constant on Eq. 2. *t* is the release time, *n* is the release exponent that depends on the release mechanism and the shape of the system tested, *kd* is the diffusional constant and *kr* the relaxational rate constant on Eq. 3. *m* is the purely Fickian diffusion exponent.

For optimal fitting of the system’s release curve, only the release data corresponding to the pH 7.5 condition was considered in the modelling.

The optimum values for the parameters present in each equation were determined using Microsoft Excel 2010 (Microsoft, Albuquerque, NM, USA). Best fitting was stated by comparing r^2^ coefficient in each of the mathematical models for the release data. Closest r^2^ to 1 better fitting.

### LpAT-S loading

As secretome loading is the interest of the 3DP core-shell tablets, after characterizing their release profile with the help of fluorescein as a traceable molecular model we determined the LpAT-S loading capacity of the formulations, the amount of secretome present in the doses of hydrogel dispensed by the printer during the process was evaluated. This way, secretome dose hydrogel ink dispensed during actual printing was collected and dissolved in PBS. After complete dissolution of the hydrogel, Interleuquin 1 receptor antagonist (IL1-Ra) ELISA (Invitrogen, Thermo Fisher Scientific, Waltham, MA, USA) was assayed for each sample. IL1-Ra was established as molecule of interest among the composition of the vehicled LpAT-S. As a control group, a fresh LpAT-S dose was employed. Experiment was performed in triplicate.

### Determination of LpAT-S degradation after drying step

In order to test whether drying stage of the 3DP core-shell tablets could affect LpAT-S proteins stability, IL1-Ra stability was tested during two different drying techniques. For such aim, secretome hydrogel ink doses were dispensed through the printer and collected for analysis. The collected hydrogel ink doses were tested by ELISA (Invitrogen) to determine the initial concentration of IL1-Ra in the formulation (*n* = 10). The remaining doses were subjected to drying conditions: Dry heat drying in an oven at 37 °C for 48 h (*n* = 10). Subsequently, the concentration of IL1-Ra available in the ink doses after drying was determined and compared to initial concentration.

### LpAT-S release and protection against acid degradation

To determine the protective capacity of the core-shell tablets against gastric pH, the concentration of the cytokine IL1-Ra was determined by ELISA (Invitrogen, Thermo Fisher Scientific, Waltham, MA, USA n) after the final disintegration of the formulations. Thus, on the one hand, 3DP core-shell tablets loaded with LpAT-S were immersed in 0.1 M HCL for 2 h with stirring at 75 x rpm. After this acid stage, the formulations were immersed in 10 mL of PBS until completely disintegrated. Afterwards, the sample was centrifuged at 2000 rpm for 10 min to pellet formulation debris and the amount of IL1-Ra present in the supernatant was determined by ELISA (Invitrogen, Thermo Fisher Scientific, Waltham, MA, USA). At the same time, the amount of IL-1Ra was determined in the supernatants of 3DP core-shell tablets disintegrated directly in 10 mL of PBS without undergoing a prior acid step. Secretome-loaded hydrogel doses undergoing the same printing and drying conditions of the 3DP core-shell tablets was employed as control group for the experiment. Experiment was performed in triplicate. IL1-Ra in each group was determined and compared in order to state LpAT-S protection capacity of the 3DP core-shell tablets.

### Statistical analysis

One-way ANOVA was employed to analyze results when normally distributed. A Bonferroni or Tamhane post-hoc analysis based on the homogeneity of variances Levene’s test was applied. Non-normally distributed data was analyzed by Mann-Whitney´s nonparametric analysis. Results were expressed as the mean ± standard deviation. Statistical analysis was executed using SPSS 25.0 (SPSS^®^) and *p* < 0.05 was considered statistically significant. For the graphical representation of the results, GraphPad Prism 5.01 (GraphPad Software, Inc.) was employed.

## Results

### Morphological appearance of the core-shell tablets

**Fig. 1:**
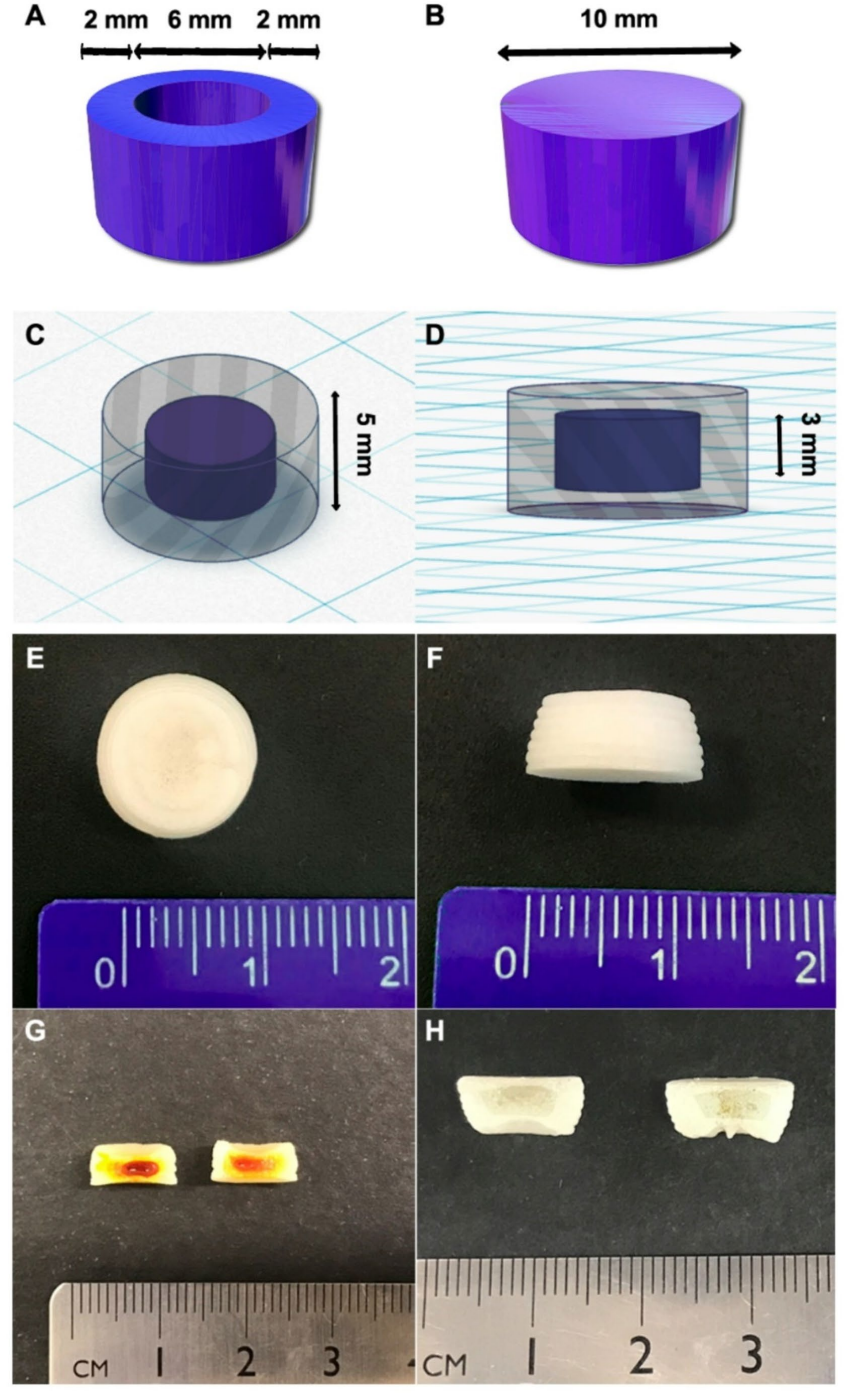
Morphological evaluation of the 3D-printed core-shell tablets. (**A, B, C, D**) Digital designs of the 3DP core-shell tablets. (**E**) Top view photograph of a 3DP core-shell tablet. (**F**) Side view of a 3DP core shell tablet. (**G**) Photograph of a 3DP core-shell tablet loaded with fluorescein split in half (**H**) Photograph of a 3DP core-shell tablet loaded with LpAT-S split in half

3D printed core-shell pills were photographed and measured in order to compare its appearance and printing accuracy. As depicted in Fig. 1, **3**DP core-shell tablets demonstrated to be similar to the digital design (Fig. 1A). Circular appearance of the printlets was observed with 10.34 ± 0.24 mm width and 5.33 ± 0.23 mm height and 5.33 ± 0.23 and 10.4 ± 0.14 mm width and 5.2 ± 0.22 mm height sizes for the fluorescein and LpAT-S loaded 3DP core-shell tablets respectively determined after drying stage (Table 3). The printlets exhibited minimal deformations in the lower layers caused by spreading due to the weight of the upper layers of the safe-shell ink. Additionally, slight central depressions in the area of the hydrogel ink hole were observed, likely due to a reduced resistance to the weight of this formulation. No gaps or holes were observed in the formulations, thus demonstrating a continuous coating of the hydrogel core.

### SEM imaging

**Fig. 2:**
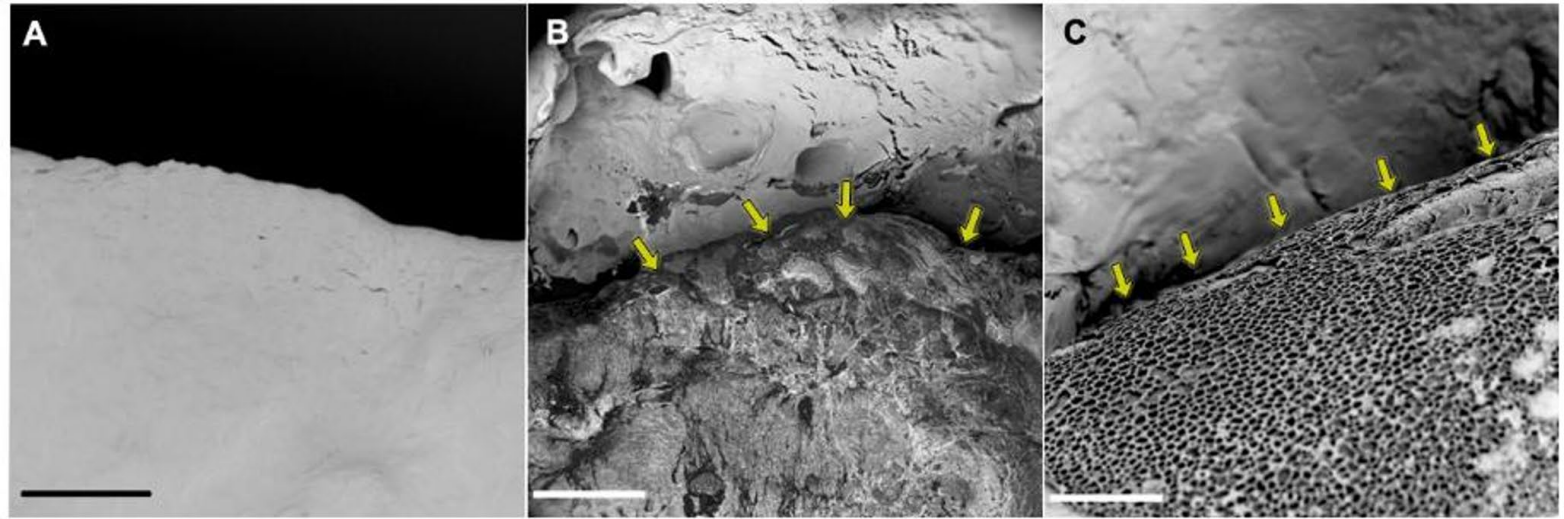
SEM imaging of the 3D-printed core-shell tablets. (**A**) SEM image of the external Eudragit shell with no gaps or voids visible (**B**) Internal part of a LpAT-S loaded 3DP core-shell tablet. Yellow arrows pointing the hydrogel core internal part. (**C**) Internal part of a non-loaded 3DP core-shell tablet. Yellow arrows pointing the hydrogel core internal part. Scale bar represents 100 μm

**Fig. 3:**
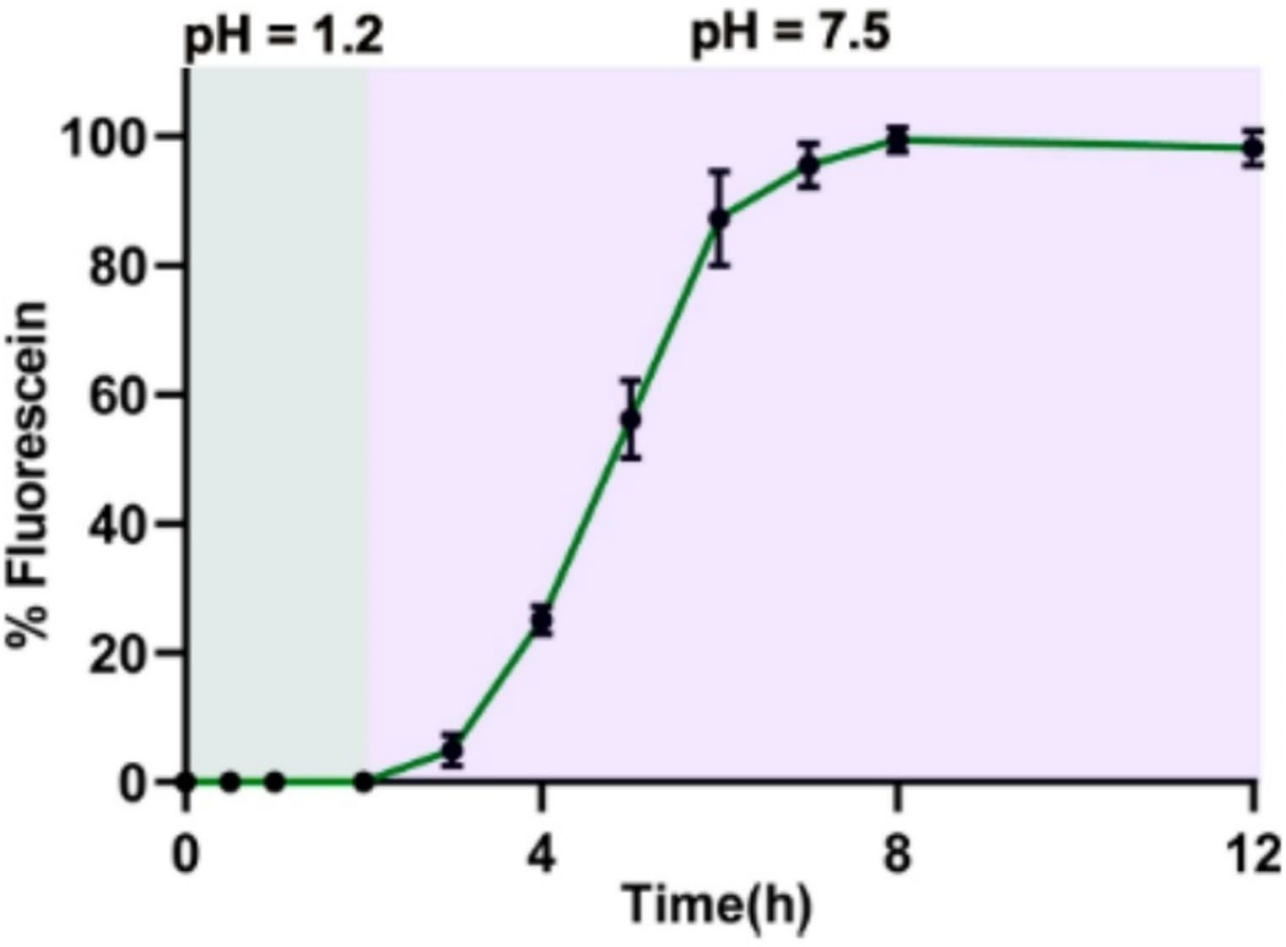
Dissolution profile of fluorescein loaded 3DP core-shell tablets. The graph illustrates the release profile of fluorescein from the 3DP core-shell tablets. The release rate was monitored to assess the effectiveness of the gastro-resistant coating and the sustained release characteristics of the tablets, demonstrating consistent release behavior in simulated gastric (green coloured zone) and intestinal fluids (pink coloured zone)

SEM imaging was employed to observe the internal structure of the different inks employed for the formulation of the 3D printed core-shell tablets. As shown in Fig. 2A, safe-shell coating layer was continuous and smooth with very few shallow cracking observed in the surface. Notably, the appearance of the hydrogel core changed when formulated with and without LpAT-S, with the formulations containing LpAT-S demonstrating a more intricate appearance (Fig. 3B, C).

### Mass variation

Weight of the printlets loaded with fluorescein was 384.66 ± 5.26 mg. Weight of the printlets loaded with LpAT-S was 387.32 ± 4.65 mg (Table 3) Mass variability fits well within the ± 5% range set by the European Pharmacopoeia for tablets with an average mass greater than 250 mg.

### Friability testing

Formulation demonstrated to lack friability. Weight change in the core-shell tablets was not observed during the test for either of the fluorescein or LpAT-S loaded 3DP core-shell tablets (Table 3).

### Hardness testing

Hardness of the dried printlets was higher than the equipment force limit in both fluorescein loaded and LpAT-S loaded formulations (Table 3).

### Humidity test

Water loss of LpAT-S loaded formulations was 2.33% (w/w) and water loss of fluorescein loaded printlets was 3.52% (w/w).

### Disintegration time

Complete disintegration of 3DP core-shell tablets was not observed during the study. However, changes in the morphology of the printlets was observed, as fragmentations and holes of the shell membrane appeared after 2 h of testing in phosphate buffer media (Table 3).

**Table 3:** Physical characterization results for the fluorescein and LpAT-S loaded 3DP core-shell tablets

	Weight (mg ± SD)	Widht (mm ± SD)	Height (mm ± SD)	Friability	Hardness (*N*)	Disintegration time (h)	Water loss (% w/w)
Fluorescein loaded	384.66 ± 5.26	10.34 ± 0.24	5.33 ± 0.23	-	> 500	> 1	3.52
Secretome loaded	387.32 ± 4.65	10.4 ± 0.14	5.2 ± 0.22	-	> 500	> 1	2.33

### Dissolution testing

Fluorescein release in acid state was not observed. After 1 h of pH 7.5 stage fluorescein was released progressively from the 3DP core-shell tablets. Maximum release was observed within 8 h of dissolution test. No burst release was observed during the study and 100% of the fluorescein content inside the printlets was released.

Mathematical fitting of the release at pH 7.5 data to zero order, Korsmeyer-peppas and Peppas Sahlin models can be observed in Table 4. As depicted, the systems shows best fitting to Peppas Sahlin´s model kinetics (r^2^ = 0.992). Results suggest that fluorescein release follows a non-Fickian mechanism. Based on the Kd (0.0316) and Kr (0.0035) values obtained from the model, the release mechanism is best described as a combination of fluorescein diffusion through the system and matrix relaxation.

Thus, with results obtained from data modelling we stated that the system studied consists of a core/shell structure, where the outer shell functions as a pH-dependent barrier that remains intact at acidic pH and dissolves at pH 7.5. After shell dissolution, the system behaves as a matrix-type drug delivery platform, which determines the subsequent release behavior. To accurately capture this transition, only the release data obtained at pH 7.5 was fitted to mathematical models, as it represents the matrix-controlled release phase. In addition, we clarified the description of the release mechanism by reporting the R² values of the model fittings: 0.987 for the Korsmeyer–Peppas model and 0.992 for the Peppas–Sahlin model. These high R² values indicate an excellent fit and support the interpretation of a non-Fickian release mechanism, involving both diffusion of fluorescein through the hydrated matrix and relaxation of the polymer network.

**Table 4:** Fluorescein release kinetics model fitting parameters. (1) zero order constant; (2) Korsmeyer constant; (n) release exponent (m) Fickian diffusion exponent; (Kd) Peppas diffusion kinetic constant; (Kr) Peppas relaxation kinetic constant.; (r^2^) determination coefficient

Zero order		Korsmeyer-peppas			Peppas Sahlin			
K^1^ (min-1)	r^2^	K^2^ (min-^n^)	n	r^2^	Kd (min^− 0.5719^)	Kr (min^− 1.1438^)	m	r^2^
0.1998	0.7592	0.0421	0.5897	0.987	0.0316	0.0035	0.5719	0.992

### LpAT-S loading

**Fig. 4:**
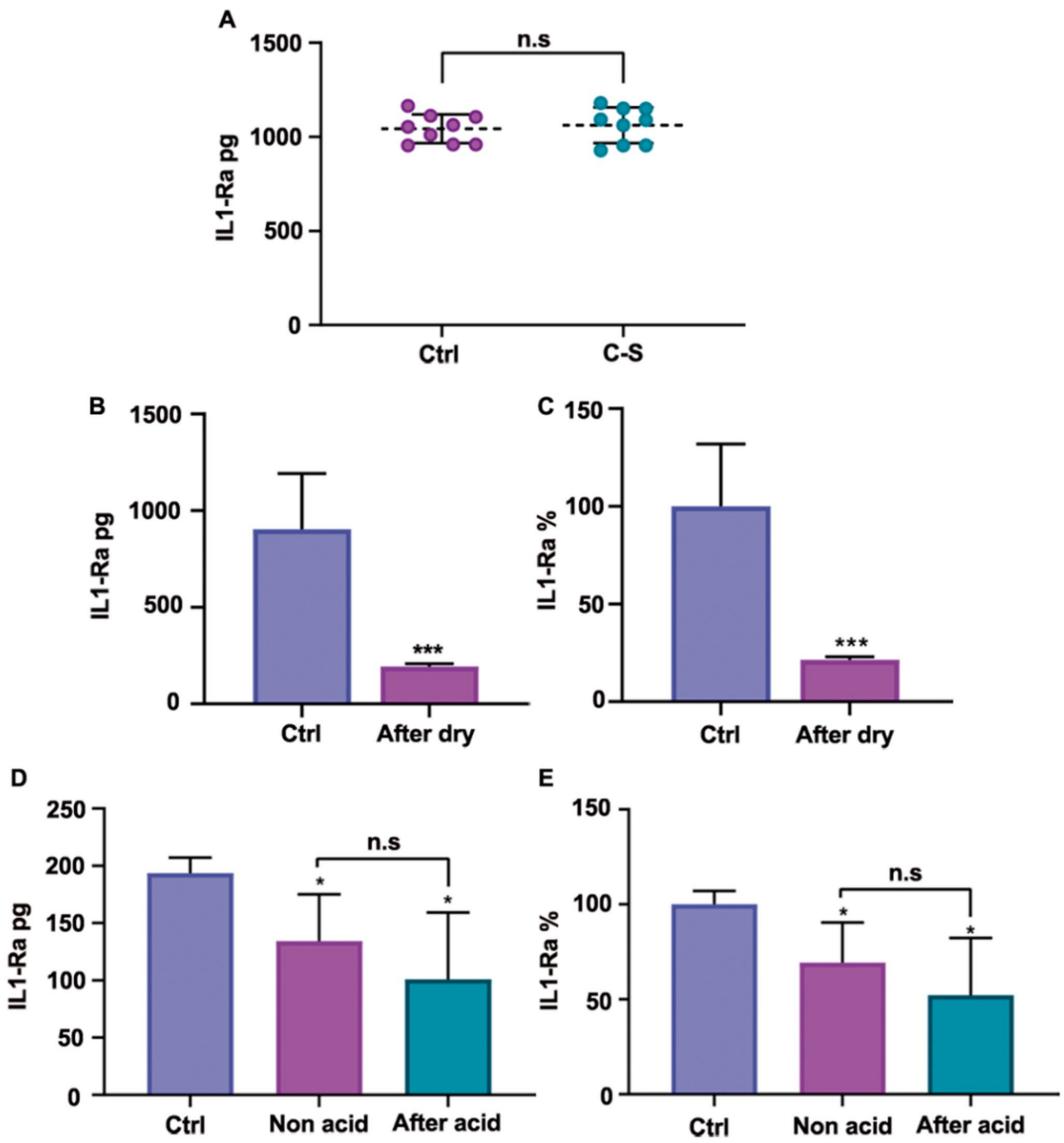
Secretome loading and release inside the 3DP core-shell tablets. (**A**) Drug loading of IL1-Ra as a representative LpAT-S molecule inside the secretome loaded hydrogel doses dispensed by the 3D-printer (C-S). Ctrl group states for LpAT-S doses without being printed. (**B**) Comparison of IL1-Ra amount inside the LpAT-S loaded 3D-printed doses after drying stage at 37 °C for 48 h against IL1-Ra amount inside the LpAT-S loaded 3D-printed doses without drying step. (**C**) Comparison of IL1-Ra percentage inside the LpAT-S loaded 3D-printed doses after drying stage at 37 °C for 48 h against IL1-Ra percentage inside the LpAT-S loaded 3D-printed doses without drying step (**D**) Comparison of IL1-Ra amount released by 3DP core-shell tablets loaded with LPAT-S after being submerged in 0.1 M HCl for 2 h and formulations that have not been submerged in HCl. The control group consists of hydrogel doses loaded with LpAT-S dispensed by the printer and outgoing drying conditions of 37 °C for 48 h. (**E**) Comparison of the percentage of IL1-Ra release of 3DP core-shell tablets loaded with LPAT-S after being submerged in 0.1 M HCl for 2 h and formulations that have not been submerged in HCl. The control group consists of hydrogel doses loaded with LpAT-S dispensed by the printer and outgoing drying conditions of 37 °C for 48 h. For all the graphics * represents comparison against Ctrl group. *p* < 0.05 *, *p* < 0.01 **, *p* < 0.001 ***, n.s Non-significance

IL1-Ra average content inside each of the 3DP core-shell tablets was 1064.20 ± 94.94 pg showing non-significant differences (*p* = 0.6350) with secretome doses control group (Fig. 4A).

### LpAT-S degradation after drying stage

Drying stage proved to degrade IL1-Ra loaded inside de secretome hydrogel dose ink. An average of 21% of the dosed IL1-Ra was maintained after drying stage.

### Secretome protection capacity

As shown in Fig. 4D/E, LpAT-S loaded 3DP core-shell tablets demonstrated the capacity of release. Release was maintained both after acid and non-acid exposure. Although total release was decreased in the formulations comparing to control group, no significant differences (*p* = 0.3869) were observed between printlets outgoing acid state previous to release (69.35% of IL1-Ra released) and printlets released without acid state (52.18% of IL1-Ra released).

## Discussion

The progress in research for advanced therapies shows the need for a paradigm shift in which existing commonly employed pharmaceutical forms are developed and adjusted to new, more sophisticated and demanding therapeutic weapons. Thus, in this process of modernization and personalization of therapies, not only the advancement but also the adjustment of the formulation processes is important. This means that many of the excipients used in conventional manufacturing processes must also be adapted to novel and emerging manufacturing processes such as 3D printing. This adaptation from the traditional to the novel goes through an optimization of the formulation process that can sometimes be complex. This study exemplifies the adaptation of excipient formulation to modern techniques. This is the case of Eudragit adaptation to semisolid extrusion 3D-printing process of coated oral dosage forms. Eudragit L100-55 is a methacrylate copolymer commonly employed for gastro resistant coating of tablets, capsules or microgranules in pharmaceutical industry [25, 26]. It is normally used as a suspension that is sprayed in the surface of the formulations and dried for coating [27]. Its formulation within a coating suspension is simple since it can be easily dispersed in aqueous media by combining it with ethanol and small amount of plasticizers such as triacetin [28]. Thus, Eudragit L100-55 coating is a well-known stablished practice in traditional pharmaceutical manufacturing processes [29]. However, coating oral formulations by spraying Eudragit suspensions in 3D printed formulations extends the manufacturing process. Therefore, the production of 3D-printed pharmaceutical forms that are subsequently coated by spraying Eudragit does not represent a real advancement for the field and makes the manufacturing process unnecessarily complex, contrary to the goal of simplifying and automating them through 3D printing techniques [30, 31].

The challenge of applying surface coatings in 3D printing processes is a significant limitation. It is complex to apply aqueous coating membranes on already printed pharmaceutical forms in a simple way, or at least to do it in the conventional way [31]. Consequently, this has paved the way for scientific innovation, leading to the development of gastro-resistant 3D printed systems utilizing Eudragit in suspension form without the need for surface spray coating [30, 32–36]. As an example, Linares et al. used the injection of Eudragit suspensions into drug loaded into porous PLA printlets to achieve a gastro-resistant coating effect [36]. Nevertheless, while this system allows an optimal coating of the formulations, it shows limitations since the specific spatial placement of Eudragit coating can be compromised. Thus, progress in 3D printing processes in which the specific placement of excipients allows control of the release profiles of formulations is limited [37–39]. Therefore, adapting Eudragit to 3D printing Pharma-inks that can be spatially placed in sites of interest and offer in situ coating during printing, without the need for post-printing processes, is of special interest.

Various researchers have used blends of Eudragit powders to obtain printed formulations via SSE techniques, employing high temperatures during the printing process [40]. While this approach yields good results, it is not suitable for all drugs and could be inappropriate for LpAT-S, which is potentially sensitive to high temperatures. Therefore, for this type of therapy, it is particularly important to develop manufacturing techniques that do not require elevated temperatures for the inks.

Other authors have successfully created coatings based on Eudragit through SSE 3D printing without rising temperature during the process, but they used formulated commercial pastes whose compositions were not disclosed [41]. This lack of transparency limits the progress of research and hampers the advancement of 3D printing technologies. Thus, is not only development what is needed but also simplicity in formulation and spreading of knowledge what is sought in this field.

Naturally, the formulation of Eudragit L100-55 into an SSE ink printable without high temperature poses several challenges that we faced during our development. First, in attempting to apply Eudragit L100-55 powder as a printable ink rather than as a sprayable suspension, we were concerned about the polymer concentration required to achieve the gastro-resistant effect. Thus, the maximum Eudragit concentration in the formulated Pharma-ink was sought to ensure the effective coating characteristics of the polymer. However, using Eudragit as the main component posed many limitations.

Briefly, Eudragit L100-55 demonstrated to be non soluble in water [25, 27]. Certainly, when mixed with water the polymer demonstrated to swallow producing an elastic paste non-suitable for being extruded. Therefore, with the objective of formulating a Eudragit paste we chose to use liquid plasticizers such as TEC or PEG to moisturize the polymer, avoiding swelling and obtaining a paste with better flow characteristics (Supplementary material 1). Nevertheless, when mixing Eudragit with plasticizers, the resulting paste consistency became too fluid to control its extrusion properly and non-controllable flow of the pastes was observed through the nozzle of the printer. In this manner, we aimed to develop a formulation capable of retaining moisture while maintaining a certain viscosity and elastic character to facilitate extrusion and shaping. In order to support water retention inside the Eudragit ink formulation we employed an HPMC suspension. HPMC is a well-known employed polymer employed in pharmaceutical industry for the formulation of semi-solid vehicles such as hydrogels [42]. When mixed with water, HPMC forms hydrogen bonds with water molecules, leading to hydration and solubilization of the polymer [43]. In our study we employed low molecular weight HPMC 10% solution which forms a viscous solution capable to retain water and moisture Eudragit without leading to polymer swelling. Water retention capacity of the HPMC solution was combined with the effect of the plasticizer TEC that demonstrated to support viscoelastic properties of the resulting paste and avoid its rapid drying enhancing its flexibility and flow capacity through the nozzle of the printer when extruded. As a result, a Eudragit L100-55 based paste (Safe shell ink) was obtained and employed as a coating 3D- printable layer during the study. Notably, this safe shell ink demonstrated to be capable of avoiding drug release in acid state, which was aligned with the objective of the study of reproducing gastro-resistant Eudragit coating in 3D printing processes. Moreover, this paste permitted easy molding through extrusion, offering excellent printability characteristics. This enabled the reproduction of digital designs, allowing the printing of hollow shapes without the structures collapsing (Fig. 4C/D). Interestingly hollow safe shell ink structures were successfully loaded with the hydrogel inks, which allowed LpAT-S formulation inside of the protective structure easily.

However, core-shell system loading was achieved thanks to an alginate based hydrogel pharma innk that enables Eudragit shell filling with secretome accurately. Alginate hydrogels are known for their ability to form ionic crosslinked networks in the presence of divalent cations such as calcium, enabling the development of systems with tunable mechanical properties and release profiles [24.25]. In this study, we employed a medium-viscosity alginate (approx. 200 mPa·s) with a high guluronic acid content (G/M ratio > 1), which is known to promote stronger and more stable gel networks. This composition was chosen to balance sufficient mechanical integrity with the need for an injectable, printable formulation. The selection of alginate was also motivated by its proven capacity to protect bioactive molecules, such as proteins present in the secretome, from harsh environments and to enable sustained release. Moreover, high water content and mild gelling conditions of alginate make it suitable for secretome delivery. Overall, the use of alginate in combination with Eudragit allowed us to design a hybrid system capable of achieving both protective and controlled release functionalities.

Notably, in terms of physical characterization, both fluorescein-loaded and LpAT-S loaded 3DP core-shell tablets showed consistent width and height measurements, closely matching their designed specifications, as shown in Table 3. Variability in the shape of printed constructs is usually expected in the SSE printing of pastes, due to their high water content leading to spreading of the bottom layers [44, 45]. However, the initial water content was shown to be removed during the manufacturing process, as the water content of neither the fluorescein nor the LpAT-S printlets was above 5% (w/w). Despite minor deviations, these formulations exhibited uniform weight distribution. Additionally, the printlets demonstrated exceptional hardness, exceeding 500 N, indicating robust physical integrity. Despite the tablets not disintegrating in the disintegration tests, their dissolution profile showed complete release of the fluorescein used as a model. Furthermore, complete disintegration of the printlets was observed in the dissolution studies. These results stated the 3DP core-shell formulations as gastro resistant non brittle and suitable printlets for oral administration.

Remarkably, not only the safe shell ink was crucial for the printing of the 3DP core-shell tablets. Incorporating LpAT-S into the alginate hydrogel ink enabled us to create concentrated, easily producible, and extrudable LpAT-S doses. Furthermore, as shown in Fig. 4A, the LpAT-S loading did not differ from the control group, indicating that the 3D-printing of the LpAT-S loaded hydrogel ink does not introduce significant variability in the dosing process. This dosing approach overcame the limitations of liquid AT-S delivery (dose limitation and high volume of the doses), allowing us to precisely control the dose loaded into the printlets by adjusting the amount of LpAT-S formulated within the hydrogel ink vehicle.

When discussing therapies such as LpAT-S, delivery devices must meet specific requirements to ensure the stability of these treatments. Briefly, LpAT-S is composed mainly by growth factors and cytokines, proteins that are sensitive to extreme temperature and pH conditions [5, 46, 47]. Thus, the need to control these features during its formulation is evident. In our study, for example, the sensitivity of the IL1-Ra protein included in the LpAT-S to drying conditions was demonstrated. IL1-Ra is a protein with a molecular weight of approximately 17.2 kDa, has been described as one of the cytokines present in AT-S and has been attributed a notable immunomodulatory effect, primarily on T cells and macrophages [48–50]. Although it is generally assumed that most secretome proteins are stable at temperatures below 37 °C, exposure to this temperature for 48 h was shown to degrade the protein by more than 70% (Fig. 4B/C).

Notably, the final obtained 3DP core-shell 3D printed formulation demonstrated to be capable to release the LpAT-S loaded after acid stage exposure (Fig. 4D/E). Thus, the system demonstrated to be able to vehicle LpAT-S orally by avoiding its gastric degradation. These were groundbreaking results stating our formulation as the first one capable of delivering secretome orally in a safe and efficient manner.

An interesting aspect to consider in this study is the adaptability of the analysis processes of formulations according to conventional pharmaceutical standards, such as pharmacopeia requirements [51, 52]. Traditional standards are not always useful for evaluating innovative pharmaceutical forms like 3D-printed ones or adapting to the needs of advanced therapies like secretome [53, 54]. Thus, for example, one of the limitations encountered during this study was the adaptation of dissolution studies of oral dosage forms to our LpAT-S loaded printlets. The high volumes of dissolution medium required during the studies were not compatible with the detection limits of ELISA methods for the detection of secretome proteins (IL1-Ra). Thus, when performing the dissolution tests in the established volumes, the protein released from the core-shell tablets was too diluted to be detected. This problem forced us to use fluorescein as an easily traceable model molecule in the dissolution tests of the printlets.

Fluorescein provided an approximation of the system’s release behavior, allowing us to establish the kinetic models of release. However, this approach is not the most applicable to a system loaded with LpAT-S, given the heterogeneity in LpAT-S composition [5, 10, 55]. The release of each molecule type of its composition might differ under real conditions, influenced by their size, shape, and charge. During our study, we confirmed the ability of the core-shell tablets to carry LpAT-S by determining the concentration of IL1-Ra at the final dissolution time, although the parameters used differed from pharmacopoeia standards. To test the core-shell tablets ability to protect LpAT-S from acid degradation, we used HCL volumes similar to those in the European pharmacopeia. However, we completed the dissolution of the formulations in low volumes (10 mL of PBS) after the acidic stage to quantify IL1-Ra released by ELISA. These adaptations are useful for testing the functionality of the systems, however they cause other limitations to take into account that can influence release results. For example, incompatibilities between LpAT-S proteins and formulation excipients are magnified in lower volumes. In fact, in our research, the pH variations caused by Eudragit in solution in such low volumes proved to be capable of degrading the proteins released from the system. Although we optimized solution volumes to minimize these interactions, the decreased release percentage observed in the results both in the after acid and non-acid group (Fig. 4C/D) was likely due to protein degradation during dissolution studies in low volumes and not the printlets release capacity. This highlights the need to adapt traditional dissolution studies for more demanding therapies like LpAT-S and makes progress in the field mandatory.

## Conclusion

In conclusion, this study offers for the first time a solid oral dosage form for the administration of secretome therapies. The formulation obtained is capable of not only transporting the secretome but also protecting it from gastric degradation. Notably, during the development of this formulation, excipients commonly used in industry such as Eudragit were adapted to the formulation of inks useful for SSE 3D printing techniques. As an advantage, the production of this formulation through 3D printing allowed the design of a formulation with a complex morphology that was compatible with secretome correct dosing and delivery. Furthermore, the production in small batches of these formulations avoids waste of the secretome. Overall, this research paves the way for further exploration into the oral administration of secretome therapies and highlights 3D printing as a promising production method.

## Supplementary Information

Below is the link to the electronic supplementary material.

Supplementary Material 1

## Data Availability

The data generated during the current study is available from the corresponding author on reasonable request.
