# Supplementary material for: 3D-Printed core-shell tablet for effective oral delivery of AT-MSC secretome in inflammatory bowel disease therapy
Source: Drug Deliv Transl Res. 2025 Aug 8;16(7):2179–93. doi: 10.1007/s13346-025-01932-7 (PMC13294233; doi:10.1007/s13346-025-01932-7)
Supplement: Supplementary file 1 — Supplementary Material 1 [file 13346_2025_1932_MOESM1_ESM.docx]

**Supplementary material**

**Supplementary material.** Outcomes on Eudragit L100-55 mixture with plasticizers Triethyl citrate (TEC) or Polyethylene glycol (PEG) of low molecular weight (PEF 400)

| **Ink name** | **Eudragit L100-55** | **TEC (%)** | **PEG (%)** | **Printing outcomes** |
| --- | --- | --- | --- | --- |
| E1T1 | 40 | 60 | - | Too fluid to shape |
| E2T2 | 50 | 50 | - | Too fluid to shape |
| E2T3 | 60 | 40 | - | Non extrudable |
| E1P1 | 40 | - | 60 | Too fluid to shape |
| E1P2 | 50 | - | 50 | Too fluid to shape |
| E1P3 | 60 | - | 40 | Too fluid to shape |
| E1P4 | 70 | - | 30 | Non extrudable |
